# Link Between the Cardiometabolic Index and Stroke Risk Across Different Glucose Metabolic Status in Chinese Middle‐Aged and Older Adults: A Longitudinal Cohort Study

**DOI:** 10.1155/ije/6169486

**Published:** 2026-10-13

**Authors:** Meng Li, Yongbo Yu, Xuan Chen, Yonglai Zhang, Wei Lu

**Affiliations:** ^1^ Department of Neurology Taiyuan City Central Hospital Peking University First Hospital Taiyuan Branch The Ninth Clinical Medical College of Shanxi Medical University Taiyuan Shanxi China; ^2^ Department of Neurology Taiyuan People’s Hospital Taiyuan China; ^3^ School of Software North University of China Taiyuan Shanxi China nuc.edu.cn; ^4^ Department of Critical Care Medicine Taizhou Municipal Hospital (Taizhou University Affiliated Municipal Hospital) Taizhou University Taizhou Zhejiang China tzc.edu.cn

**Keywords:** cardiometabolic index, CHARLS, glucose metabolic status, insulin resistance, stroke

## Abstract

**Background:**

The cardiometabolic index (CMI) encompasses central obesity and atherogenic dyslipidemia, two metabolic abnormalities that are closely associated with insulin resistance and issues in glucose metabolism. However, whether the association between CMI and incident stroke differs across glucose metabolic states remains unclear.

**Methods:**

Utilizing data from the China Health and Retirement Longitudinal Study (CHARLS), we carried out a cohort study with 7980 individuals aged 45 years and above who were free of stroke. We explored the associations between the initial CMI and stroke incidents across various glucose metabolism status through Cox proportional hazards models, restricted cubic spline analyses, Kaplan–Meier survival analysis, subgroup and sensitivity analyses, and receiver operating characteristic analysis.

**Results:**

Incident stroke risk was independently associated with both log (CMI) and CMI quartiles, increasing with higher values and quartiles. The fully adjusted model showed that each 1‐unit rise in log (CMI) was associated with an increased risk of stroke (HR = 1.13, 95% CI 1.00–1.28; *p* = 0.044). Compared to Q1, Q4 had a higher risk of stroke (HR = 1.52; 95% CI 1.11–2.08; *p* = 0.008). Moreover, for individuals with diabetes mellitus (DM), a higher CMI was associated with a greater likelihood of stroke, with Q4 presenting an increased risk (HR = 1.49, 95% CI: 1.02–2.31, *p* = 0.019) when compared to Q1. By contrast, no significant association was observed among individuals with prediabetes or normal glucose regulation. The findings’ robustness was further corroborated by conducting subgroup and sensitivity analyses. In those with DM, the CMI had a moderate level of accuracy in predicting new stroke cases, with an area under the receiver operating characteristic curve of 0.675.

**Conclusions:**

A higher baseline CMI was associated with an increased risk of incident stroke, with a more apparent association among individuals with DM. These findings suggest that the vascular relevance of the CMI may vary according to the underlying glucose metabolic status and support its potential role as a marker of cardiometabolic dysfunction. Further research is essential to shed light on the metabolic pathways involved in these associations and to establish the clinical value of CMI.

## 1. Introduction

Stroke is characterized by a sudden interruption or reduction in cerebral blood flow, leading to localized or widespread neurological damage. Strokes are divided into ischemic and hemorrhagic categories, with ischemic strokes accounting for about 84.4% [1]. With more than 13.7 million strokes occurring each year, they are the second largest cause of death globally, resulting in 5.5 million fatalities and extremely high morbidity, disability, and mortality rates [2]. Since 2015, stroke has emerged as the foremost cause of death and disability in China. Being a chronic noncommunicable disease, it poses a significant health risk to China’s population [3]. Consequently, identifying high‐risk stroke groups early and applying efficient preventive measures is one of the most critical challenges right now [4].

The occurrence of strokes is highly correlated with glucose metabolism disorders, which are also risk factors for cardiovascular events [5, 6]. The risk of stroke is considerably higher in individuals with glucose regulation problems, including those with prediabetes (Pre‐DM) and diabetes mellitus (DM) [7]. Those classified as having normal glucose regulation (NGR), despite not being diabetic, may experience subtle metabolic imbalances that raise their stroke risk, suggesting that glucose metabolism spans a range that calls for more thorough risk analysis [8]. Significant progress has been made in understanding the connection between hyperglycemia and stroke, yet the exact nature of this relationship, particularly across different glucose metabolic states, remains not fully understood.

Recently, various metabolic markers, such as the triglyceride (TG)‐glucose index (TyG) [9, 10] and lipid accumulation product (LAP) [11], have been studied to evaluate stroke risk. Nonetheless, these indices show different aspects of metabolic dysfunction and may not fully reflect the combined effects of central adiposity and atherogenic dyslipidemia. In 2015, the cardiometabolic index (CMI) was proposed by Wakabayashi and Daimon, integrating the waist‐to‐height ratio (WHtR) with the TG‐to‐high density lipoprotein cholesterol ratio (TG/HDL‐C) [12]. First introduced to serve as a diabetes risk indicator [13], the CMI has been subsequently associated with cardiovascular disease, metabolic syndrome, hypertension, and other cardiometabolic disorders [14–18]. Whereas WHtR reflects central adiposity without directly capturing metabolic abnormalities [19], and TyG primarily reflects glucose–lipid metabolic dysfunction without accounting for central adiposity [20, 21], the CMI integrates the WHtR with the TG/HDL‐C ratio, thereby capturing both central adiposity and atherogenic dyslipidemia. The CMI may therefore provide a more comprehensive assessment of cardiometabolic risk by integrating central adiposity and atherogenic dyslipidemia [22]. Recognizing that central obesity, dyslipidemia, and glucose metabolism impairment often occur together, we investigated if the association between the CMI and stroke incidence differed across NGR, Pre‐DM, and DM.

By utilizing data from the China Health and Retirement Longitudinal Study (CHARLS), this research examined how the CMI, integrating central obesity and atherogenic dyslipidemia, is associated with the incidence of stroke in Chinese adults aged 45 years and older. It further aimed to analyze whether this association differed across various glucose metabolic status (NGR, Pre‐DM, DM), characterize the dose‐response relationship between CMI and stroke risk, and assess CMI’s potential to identify those at higher stroke risk.

## 2. Methods

### 2.1. Study Participants

We engaged in a prospective cohort study with longitudinal data obtained from CHARLS, a nationally representative source. Starting in 2011, CHARLS recruited individuals who were 45 years old or older and carried out interviews every two to three years thereafter (2013, 2015, 2018, 2020). Of the 17,705 respondents at baseline in 2011, we excluded individuals with (i) baseline stroke exclusion and baseline stroke absence (*n* = 594), (ii) missing data required to calculate CMI (*n* = 7725), (iii) missing fasting plasma glucose (FPG) data (*n* = 944), (iv) missing glycated hemoglobin (HbA1c) data (*n* = 61), (v) a history of cancer (*n* = 121), (vi) age < 45 years (*n* = 159), and (vii) missing follow‐up stroke outcome data (*n* = 121) (Figure 1). The ultimate analysis included 7980 adults. The follow‐up for participants started with the 2011 baseline interview and continued until they experienced a stroke, completed their final interview in 2020, were lost to follow‐up, or passed away.

**FIGURE 1:**
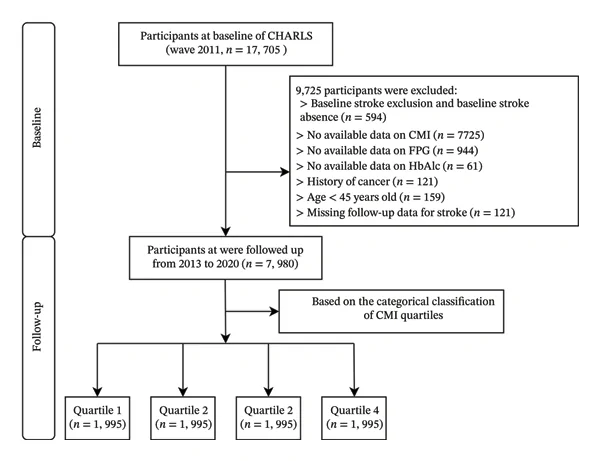
The flowchart of study participants. Abbreviations: CMI, cardiometabolic index; FPG, fasting plasma glucose; HbA1c, hemoglobin A1c. *Note:* log (CMI) denotes the log‐transformed CMI and was used in the continuous‐variable analyses; CMI quartiles (Q1–Q4) were defined using the original, untransformed CMI.

### 2.2. Exposure Assessment

In 2011, during the initial visit, measurements were conducted by trained field personnel using calibrated tools and standardized methods. To measure waist circumference, a nonelastic tape was used to the nearest 0.1 cm at the midpoint between the bottom edge of the last rib and the iliac crest, with participants standing in light clothing and gently exhaling. In cases where the first two measurements had a discrepancy greater than 1.0 cm, a third measurement was made, and the average of the two nearest measurements was taken. Height was recorded without shoes using a stadiometer with 0.1 cm accuracy, and the participant’s head was aligned in the Frankfort plane. Collections were scheduled for the morning, and fasting status was noted during the blood draw. Baseline blood collection involved taking fasting venous blood after an overnight fast of 8 h or more.

Serum TG (TG) levels were quantified in the laboratory through enzymatic colorimetric methods, and HDL‐C was measured using direct homogeneous assays as instructed by the manufacturer in a certified central lab. The laboratory used internal quality‐control materials in each run and participated in external proficiency testing; batch effects were monitored, and assay calibration was performed in line with the lab’s standard procedures. Before TG/HDL‐C was calculated, units were adjusted to mg/dL, and distributions were inspected for unlikely values; when duplicate anthropometric readings were available, the mean was used as mentioned above.

As the primary exposure measure, the CMI is defined as the product of the WHtR and the TG/HDL‐C ratio [12]. Trained staff in 2011 used standardized methods to obtain anthropometric data, calculating the WHtR by dividing the waist circumference (cm) by the standing height (cm). Fasting venous blood samples were collected during the same visit and analyzed in a central lab to determine serum TGs and HDL cholesterol levels, with results given in mg/dL. The TG/HDL‐C ratio was computed, and the CMI was calculated as the WHtR multiplied by TG/HDL‐C. During the primary analyses, log (CMI) was used as a continuous variable, while the original CMI was categorized into baseline quartiles (Q1–Q4), with Q1 as the reference point.

### 2.3. Follow‐Up of Endpoint Events

Self‐reported strokes diagnosed by physicians during follow‐up interviews are typically used in large aging studies such as CHARLS and HRS, and this method has been applied in earlier analyses with identical question wording [23–25]. Validation studies in similar groups reveal acceptable performance in identifying stroke events and estimating risk‐factor associations, although they admit to less‐than‐perfect sensitivity and positive predictive value [23, 25].

Incident stroke was the outcome, defined as the first instance of a stroke diagnosed by a physician and self‐reported during any follow‐up interview after the baseline. Standardized questions were posed to the participants by trained personnel, including “Has a medical professional told you that you have had a stroke?”; “Have you been diagnosed with a stroke by a doctor since your last follow‐up?”; and “Has there been any change in your stroke since your last interview: better, worse, or unchanged?” By asking participants “When did you first find out about your stroke diagnosis?” and “When did your most recent stroke happen?”, the timing of the stroke events was established. Participants with a history of stroke at the beginning were not part of the risk set. Follow‐up commenced in 2011, with additional interviews held in 2013, 2015, 2018, and 2020. The date of an incident stroke was assigned to the interview when it was first noted; if not, participants were censored at their last interview completed by 2020, and person‐time was monitored from the start to the event or censoring.

### 2.4. Covariate Selection

The choice of covariates was informed by clinical context and prior research evidence [26–28]. Health‐related variables and sociodemographic factors were both included in this study. Included in the sociodemographic variables were age, sex, marital status (either married/cohabiting or other), and education level (junior high school or less, senior high school, college or above). Health‐related elements comprised history of hypertension, history of dyslipidemia, history of heart disease, obesity status (normal weight, overweight, obesity), smoking status (never, former, current), and drinking status (never, former, current) [28]. Baseline data collection involved in‐person interviews with a structured questionnaire. The laboratory conducted assays on baseline fasting venous blood for HDL‐C, LDL‐C, total cholesterol, TG, FPG, HbA1c, serum creatinine, blood urea nitrogen, and uric acid, and calculated estimated glomerular filtration rate (eGFR) from serum creatinine [28]. According to the criteria established by the Working Group on Obesity in China, normal weight was defined as a BMI < 24.0 kg/m^2^, overweight as a BMI of 24.0–27.9 kg/m^2^, and obesity as a BMI ≥ 28.0 kg/m^2^, based on national evidence linking BMI to cardiometabolic risk and validated in pooled cohort studies [29, 30].

### 2.5. Data Collection

Information on demographics and health status was collected via a standardized questionnaire administered by a professional investigator. Once the participant had rested for 15 min, the investigator used a sphygmomanometer to measure blood pressure on the left arm, taking three readings spaced 45 s apart. To determine the last blood pressure value, the three measurements were averaged. Before being weighed and having their height taken, participants were told to use the bathroom and take off their jackets, shoes, hats, and socks. Height and weight were measured using standardized equipment with a precision of 0.1 cm and 0.1 kg, respectively. A qualified healthcare professional obtained a venous blood sample from each participant, which was then analyzed for a range of biochemical parameters. Hypertension was recognized by a doctor’s diagnosis, the use of medications to lower blood pressure, and/or a systolic blood pressure of 140 mmHg or higher, and/or a diastolic blood pressure of 90 mmHg or higher [31]. Dyslipidemia was characterized by a doctor’s diagnosis, the ongoing use of medications to lower lipids, and/or total cholesterol levels of 240 mg/dL or higher, TGs of 150 mg/dL or higher, HDL‐C levels under 40 mg/dL, or LDL‐C levels of 160 mg/dL or higher. The identification of DM involved a doctor’s diagnosis, the ongoing use of glucose‐lowering medications, or an FPG level of 7.0 mmol/L or more. FPG levels of 5.6–6.9 mmol/L and/or HbA1c levels of 5.7%–6.4% suggested Pre‐DM. NGR people were those who neither had DM nor Pre‐DM [31]. Moreover, self‐reported medical conditions or related treatments and issues were utilized to detect further chronic diseases [31].

### 2.6. Statistical Analysis

Testing revealed that all continuous type variables in this study did not follow a normal distribution. The description of non‐normally distributed data involved medians and quartiles, and categorical variables were detailed with the number of cases and percentages. The analysis of non‐normal continuous variables across the four groups involved the Kruskal–Wallis rank sum test, and categorical data were evaluated using the chi‐square test. Due to the non‐normal distribution of the CMI, a logarithmic transformation was conducted to enhance the distribution characteristics and fit of the model in continuous‐variable analyses. During the primary Cox proportional hazards regression analyses, log (CMI) was modeled continuously, while the original CMI was categorized into quartiles (Q1–Q4). Missing data were minimal before imputation: 1.23% for diastolic blood pressure, 1.03% for systolic blood pressure, 1.00% for dyslipidemia, 0.73% for heart rate, and less than 0.5% for all other variables (Supporting Table 1). Assuming data were missing at random, we imputed covariates using multiple imputation by chained equations (mice, R). All analytic covariates, in addition to auxiliary variables, were included in the imputation model. Five datasets were generated through imputation, with each one having 50 iterations to ensure convergence. Continuous variables were handled using predictive mean matching (“pmm”), binary variables with logistic regression (“logreg”), nominal categorical variables with multinomial models, and ordinal variables with proportional‐odds models. The verification of convergence was done using trace plots of the means and standard deviations. Cox proportional hazards regression was used to analyze each imputed dataset, and Rubin’s rules were applied to merge the results, yielding final estimates and standard errors.

Cox proportional hazards regression was performed to evaluate the association between the CMI and stroke incidence, with hazard ratios (HRs) and 95% confidence intervals (CIs) reported. Using biological plausibility and previous research, covariates were specified ahead of time. By employing scaled Schoenfeld residuals, the proportional hazards assumption was assessed for the overall model and each covariate, and no significant violations were found (*p* > 0.05 for all covariates). The evaluation of multicollinearity was conducted using variance inflation factors (VIFs) from the design matrix, and since all VIFs were under 3, no problematic collinearity was detected. In order to address confounding, three models were fitted consecutively. To be specific, Model 1 had no adjustments; Model 2 was adjusted for sociodemographic and behavioral factors (age, sex, education level, marital status, smoking status, and drinking status); and Model 3 further adjusted for clinical factors (history of hypertension, history of heart disease, history of dyslipidemia, obesity, heart rate, blood urea nitrogen, and eGFR) and glucose metabolism status. Further analysis of the link between CMI and stroke risk was conducted in subgroups defined by sex, age (under 60, 60 or older), BMI (less than 24, 24 or more), history of hypertension, GMS (Pre‐DM, DM, NGR), and history of heart disease. Using multiplicative interaction terms, the interaction of each subgroup variable with CMI was analyzed, and a forest plot was developed. Restricted cubic spline (RCS) analysis was used to investigate the potential nonlinear link between log (CMI) and stroke risk. Based on the lowest Akaike information criterion (AIC), four knots were chosen, and the RCS curves were fitted after adjusting for the previously mentioned confounders. To estimate the average number of strokes in CMI subgroups, the Kaplan–Meier approach was employed, and differences among groups were tested using the log‐rank test. To confirm the robustness of the results, we performed two sensitivity analyses: (i) repeating the models without imputing missing data and (ii) excluding stroke events that happened within 2 years of the baseline to mitigate potential reverse causation. Receiver operating characteristic analysis was utilized to verify the predictive significance of CMI for stroke risk across different stages of glucose metabolism. Receiver operating characteristic (ROC) curve analysis was used to assess the discriminatory power of CMI for incident stroke among different glucose metabolic status (NGR, Pre‐DM, and DM). The discriminatory performance of CMI was further compared with that of WHtR and TG/HDL‐C based on the area under the ROC curve (AUC) [32]. R (Version 4.4.1) and RStudio (Version 1.3.1093) were used for all statistical analyses. In this research, a two‐sided *p* value of 0.05 or below was regarded as statistically significant.

## 3. Results

### 3.1. Characteristics of Participants

Participants’ baseline demographic and clinical characteristics are sorted by CMI quartiles in Table 1. The median CMI for the whole group was 2.38, with an interquartile range between 1.43 and 4.16. Throughout the follow‐up period, there were 474 stroke incidents, equating to a total incidence rate of 5.94%. From Q1 to Q4, stroke incidence rose progressively across CMI quartiles, starting at 3.76% and reaching 7.67%, with a trend significance of *p* < 0.001. Of the 7980 individuals who participated, 46.23% were men (3,689) and 53.77% were women (4,291). Significant differences were observed in sex distribution across CMI quartiles (*p* < 0.001). For the overall population, the median age was 59.0 years, with an interquartile range of 52.0–66.0. The median age, ranging from 58.0 to 59.0 years across CMI quartiles, showed a statistically significant difference between the groups (*p* = 0.010). Significant differences were observed across CMI quartiles for all baseline characteristics (all *p* < 0.05), except for marital status (*p* = 0.111) and education level (*p* = 0.086).

**TABLE 1:** Baseline characteristics of participants categorized by CMI quartiles.

Characteristic	Overall	Quartiles of CMI				*p* value^∗^
		Q1	Q2	Q3	Q4	
No. of participants	*N* = 7980	*N* = 1995	*N* = 1995	*N* = 1995	*N* = 1995	
CMI, median (Q1, Q3)	2.38 (1.43, 4.16)	1.05 (0.83, 1.24)	1.86 (1.64, 2.13)	3.10 (2.70, 3.57)	6.35 (5.05, 9.54)	< 0.001
Age, median (Q1, Q3)	59.00 (52.00, 66.00)	59.00 (53.00, 67.00)	58.00 (52.00, 66.00)	59.00 (52.00, 66.00)	58.00 (52.00, 64.00)	0.010
SBP, median (Q1, Q3)	127.33 (114.83, 142.33)	123.67 (111.33, 138.00)	125.00 (113.33, 139.67)	129.33 (116.00, 144.33)	131.33 (119.00, 146.33)	< 0.001
DBP, median (Q1, Q3)	75.00 (67.33, 83.00)	72.67 (65.00, 80.33)	73.67 (66.33, 82.00)	75.67 (68.33, 84.00)	77.33 (70.00, 86.00)	< 0.001
Heart rate, beats/min, median (Q1, Q3)	72.00 (65.00, 79.00)	71.00 (64.00, 78.00)	72.00 (65.00, 79.00)	72.00 (66.00, 79.00)	73.00 (67.00, 81.00)	< 0.001
Gender, *n* (%)						< 0.001
Male	3689.00 (46.23%)	1103.00 (55.29%)	936.00 (46.92%)	844.00 (42.31%)	806.00 (40.40%)	
Female	4291.00 (53.77%)	892.00 (44.71%)	1059.00 (53.08%)	1151.00 (57.69%)	1189.00 (59.60%)	
Marital status, *n* (%)						0.111
Married and living with spouse	6971.00 (87.36%)	1729.00 (86.67%)	1744.00 (87.42%)	1726.00 (86.52%)	1772.00 (88.82%)	
Other	1009.00 (12.64%)	266.00 (13.33%)	251.00 (12.58%)	269.00 (13.48%)	223.00 (11.18%)	
Education level, *n* (%)						0.086
Junior high school and below	5635.00 (70.61%)	1447.00 (72.53%)	1418.00 (71.08%)	1399.00 (70.13%)	1371.00 (68.72%)	
Senior high school	2235.00 (28.01%)	526.00 (26.37%)	545.00 (27.32%)	574.00 (28.77%)	590.00 (29.57%)	
College and above	110.00 (1.38%)	22.00 (1.10%)	32.00 (1.60%)	22.00 (1.10%)	34.00 (1.70%)	
Smoking status, *n* (%)						< 0.001
Never smoked	4868.00 (61.00%)	1085.00 (54.39%)	1193.00 (59.80%)	1298.00 (65.06%)	1292.00 (64.76%)	
Currently smoking	2431.00 (30.46%)	728.00 (36.49%)	650.00 (32.58%)	536.00 (26.87%)	517.00 (25.91%)	
Ever smoked	681.00 (8.53%)	182.00 (9.12%)	152.00 (7.62%)	161.00 (8.07%)	186.00 (9.32%)	
Drinking status, *n* (%)						< 0.001
Never drink	5572.00 (69.82%)	1226.00 (61.45%)	1406.00 (70.48%)	1461.00 (73.23%)	1479.00 (74.14%)	
Currently drinking	2003.00 (25.10%)	672.00 (33.68%)	491.00 (24.61%)	418.00 (20.95%)	422.00 (21.15%)	
Ever drunk	405.00 (5.08%)	97.00 (4.86%)	98.00 (4.91%)	116.00 (5.81%)	94.00 (4.71%)	
Heart disease, *n* (%)						< 0.001
No	7048.00 (88.32%)	1808.00 (90.63%)	1791.00 (89.77%)	1766.00 (88.52%)	1683.00 (84.36%)	
Yes	932.00 (11.68%)	187.00 (9.37%)	204.00 (10.23%)	229.00 (11.48%)	312.00 (15.64%)	
Hypertension, *n* (%)						< 0.001
No	4752.00 (59.55%)	1392.00 (69.77%)	1308.00 (65.56%)	1096.00 (54.94%)	956.00 (47.92%)	
Yes	3228.00 (40.45%)	603.00 (30.23%)	687.00 (34.44%)	899.00 (45.06%)	1039.00 (52.08%)	
Dyslipidemia, *n* (%)						< 0.001
No	7203.00 (90.26%)	1911.00 (95.79%)	1854.00 (92.93%)	1793.00 (89.87%)	1645.00 (82.46%)	
Yes	777.00 (9.74%)	84.00 (4.21%)	141.00 (7.07%)	202.00 (10.13%)	350.00 (17.54%)	
Obesity status, *n* (%)						< 0.001
Normal weight	4741.00 (59.41%)	1672.00 (83.81%)	1365.00 (68.42%)	1029.00 (51.58%)	675.00 (33.83%)	
Overweight	2332.00 (29.22%)	280.00 (14.04%)	515.00 (25.81%)	703.00 (35.24%)	834.00 (41.80%)	
Obesity	907.00 (11.37%)	43.00 (2.16%)	115.00 (5.76%)	263.00 (13.18%)	486.00 (24.36%)	
GSM, *n* (%)						< 0.001
Pre‐DM	3222.00 (40.38%)	1006.00 (50.43%)	925.00 (46.37%)	799.00 (40.05%)	492.00 (24.66%)	
DM	3815.00 (47.81%)	846.00 (42.41%)	914.00 (45.81%)	968.00 (48.52%)	1087.00 (54.49%)	
NGR	943.00 (11.82%)	143.00 (7.17%)	156.00 (7.82%)	228.00 (11.43%)	416.00 (20.85%)	
WHtR, median (Q1, Q3)	0.54 (0.49, 0.58)	0.49 (0.46, 0.53)	0.53 (0.49, 0.57)	0.55 (0.51, 0.60)	0.58 (0.54, 0.62)	< 0.001
Waist circumference, median (Q1, Q3)	84.60 (78.00, 92.00)	78.20 (73.40, 84.00)	82.80 (77.00, 89.00)	87.00 (80.20, 93.20)	91.60 (85.30, 98.00)	< 0.001
HDL‐C, mg/dL, median (Q1, Q3)	49.48 (40.59, 60.31)	65.34 (57.22, 75.00)	53.74 (47.17, 60.70)	46.39 (40.98, 52.19)	37.11 (31.70, 42.91)	< 0.001
HbA1c, median (Q1, Q3)	5.20 (4.90, 5.40)	5.10 (4.80, 5.40)	5.10 (4.90, 5.40)	5.20 (4.90, 5.50)	5.20 (5.00, 5.60)	< 0.001
TC, mg/dL, median (Q1, Q3)	191.37 (168.17, 216.50)	185.95 (165.46, 209.15)	187.11 (163.92, 211.47)	192.14 (169.72, 216.50)	201.03 (175.90, 229.25)	< 0.001
TG, mg/dL, median (Q1, Q3)	103.54 (74.34, 150.45)	61.06 (52.22, 72.57)	87.61 (76.11, 101.78)	122.13 (105.32, 140.71)	201.78 (163.73, 269.92)	< 0.001
LDL‐C, mg/dL, median (Q1, Q3)	115.21 (94.33, 138.02)	108.25 (90.46, 129.12)	115.98 (96.65, 138.40)	122.17 (101.29, 143.82)	114.43 (88.53, 139.95)	< 0.001
FPG, mg/dL, median (Q1, Q3)	102.24 (94.50, 112.50)	99.00 (91.98, 107.28)	100.26 (93.42, 108.54)	101.88 (94.86, 111.60)	108.90 (99.36, 124.20)	< 0.001
eGFR, median (Q1, Q3)	94.35 (83.69, 102.04)	95.43 (86.13, 102.60)	94.81 (84.66, 102.44)	93.97 (82.90, 101.78)	92.98 (80.84, 101.28)	< 0.001
Serum creatinine, mg/dL, median (Q1, Q3)	0.76 (0.66, 0.88)	0.76 (0.66, 0.87)	0.76 (0.64, 0.87)	0.75 (0.64, 0.89)	0.77 (0.66, 0.89)	0.005
Blood urea nitrogen, mg/dL, median (Q1, Q3)	15.15 (12.58, 18.21)	16.02 (13.16, 19.33)	15.18 (12.66, 18.26)	14.73 (12.35, 17.81)	14.65 (12.30, 17.34)	< 0.001
Stroke incident, *n* (%)	474.00 (5.94%)	75.00 (3.76%)	116.00 (5.81%)	130.00 (6.52%)	153.00 (7.67%)	< 0.001

*Note:* Log (CMI) denotes the log‐transformed CMI and was used in the continuous‐variable analyses; CMI quartiles (Q1–Q4) were defined using the original, untransformed CMI.

Abbreviations: CMI, cardiometabolic index; DBP, diastolic blood pressure; DM, diabetes mellitus; eGFR, estimated glomerular filtration rate; FPG, fasting plasma glucose; GMS, glucose metabolic states; HbA1c, hemoglobin A1c; HDL‐C, high‐density lipoprotein cholesterol; LDL‐C, low‐density lipoprotein cholesterol; NGR, normal glucose regulation; Pre‐DM, prediabetes mellitus; SBP, systolic blood pressure; TC, total cholesterol; TG, triglyceride; WHtR, waist‐to‐height ratio.

^∗^Kruskal–Wallis rank sum test; Pearson’s chi‐squared test; Data were presented as mean ± SD, median and interquartile range, or as *n* (%).

### 3.2. Association of CMI With Incident Stroke

According to Table 2, log (CMI) is positively associated with the frequency of strokes. In the fully adjusted Model 3, each unit rise in log (CMI) was tied to a 13% higher likelihood of stroke (HR = 1.13, 95% CI: 1.00–1.27, *p* = 0.047). CMI’s significant association with stroke incidence persisted when examined through quartiles. Participants in Q2, Q3, and Q4 exhibited increased stroke risks compared to Q1, as shown by Model 3, with HRs of 1.50 (95% CI 1.11–2.01, *p* = 0.007), 1.47 (95% CI 1.09–1.98, *p* = 0.011), and 1.50 (95% CI 1.11–2.04, *p* = 0.009), respectively.

**TABLE 2:** The HR (95% CI) of stroke according to CMI in the three models.

Characteristic	*N*	Event *N*	Model 1			Model 2			Model 3		
			HR	95% CI	*p* value	HR	95% CI	*p* value	HR	95% CI	*p* value
Log (CMI)	7980	474	1.06	1.00, 1.12	0.045	1.34	1.21, 1.49	< 0.001	1.13	1.00, 1.27	0.047
Quartile	7980	474									
Q1	1995	75	Reference	Reference		Reference	Reference		Reference	Reference	
Q2	1995	116	1.57	1.17, 2.09	0.002	1.61	1.21, 2.16	0.001	1.50	1.11, 2.01	0.007
Q3	1995	130	1.76	1.32, 2.34	< 0.001	1.82	1.37, 2.42	< 0.001	1.47	1.09, 1.98	0.011
Q4	1995	153	2.08	1.58, 2.75	< 0.001	2.20	1.66, 2.91	< 0.001	1.50	1.11, 2.04	0.009
*p* for trend					< 0.001			< 0.001			0.028

*Note:* Log (CMI) denotes the log‐transformed CMI and was used in the continuous‐variable analyses; CMI quartiles (Q1–Q4) were defined using the original, untransformed CMI. Model 1: unadjusted; Model 2: adjusted for age, gender, education level, marital status, drinking status, smoking status; Model 3: Model 2 + adjusted for adjusted for history of hypertension, history of dyslipidemia, history of heart disease, obesity, heart rate, BUN, eGFR, GSM.

Abbreviations: BUN, blood urea nitrogen; CI: confidence interval; CMI, cardiometabolic index; DM, diabetes mellitus; eGFR, estimated glomerular filtration rate; GMS, glucose metabolic states; HR, hazard ratio; NGR, normal glucose regulation; Pre‐DM, prediabetes mellitus.

Upon controlling for confounding factors (Figure 2(A)), a significant nonlinear correlation between log (CMI) and stroke risk was observed (*p* for overall association = 0.014; *p* for nonlinearity = 0.023). Higher log (CMI) values were associated with an increased stroke risk, but this association became weaker as log (CMI) approached 1. Kaplan–Meier cumulative incidence curves in Figure 3(A) were employed to assess the connection between CMI and stroke occurrences. The data revealed a statistically significant and consistent growth in stroke incidence from Q1 to Q4 across the quartiles (*p* < 0.0001).

**FIGURE 2:**
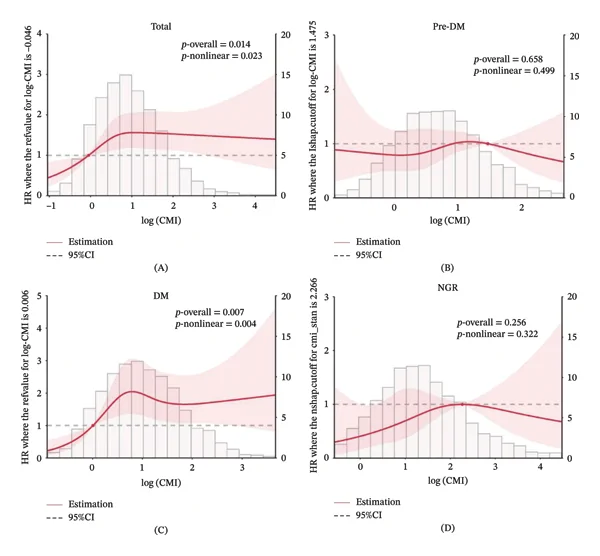
Restricted cubic spline analysis of the association between log (CMI) and incident stroke. (A) Total participants; (B) participants with Pre‐DM. (C) Participants with DM; (D) participants with NGR. *Note:* Log (CMI) denotes the log‐transformed CMI and was used in the continuous‐variable analyses; CMI quartiles (Q1–Q4) were defined using the original, untransformed CMI. Abbreviations: CI, confidence interval; CMI, cardiometabolic index; DM, diabetes mellitus; HR, hazard ratio; NGR, normal glucose regulation; Pre‐DM, prediabetes mellitus.

**FIGURE 3:**
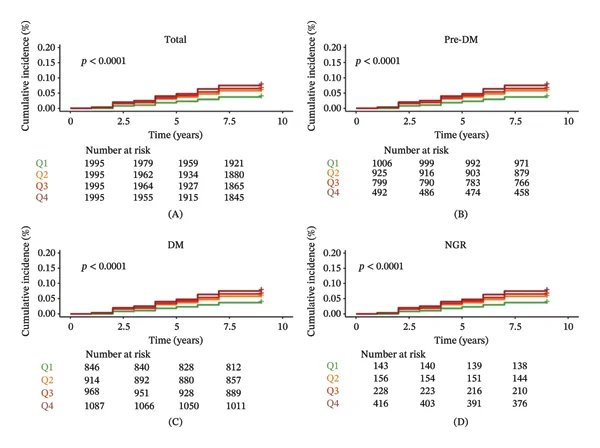
The Kaplan–Meier analysis for stroke was based on CMI quartiles. (A) Total participants; (B) participants with Pre‐DM. (C) Participants with DM; (D) participants with NGR. *Note:* Log (CMI) denotes the log‐transformed CMI and was used in the continuous‐variable analyses; CMI quartiles (Q1–Q4) were defined using the original, untransformed CMI. Abbreviations: CMI, cardiometabolic index; DM, diabetes mellitus; NGR, normal glucose regulation; Pre‐DM, prediabetes mellitus.

### 3.3. Relationship Between CMI and Stroke Risk Across Different Glucose Metabolic States

According to Table 3, the study explored how stroke incidence varied with different glucose metabolic conditions over the follow‐up period. In the Pre‐DM cohort of 3222 individuals, 151 strokes were documented. The DM cohort, consisting of 3815 individuals, recorded 246 strokes. Meanwhile, the NGR group, which included 943 individuals, reported 77 strokes. In Models 1 and 2, the association between log (CMI) and stroke risk for participants with DM notably remained significant. Specifically, Model 1 showed that a higher log (CMI) correlated with a heightened stroke risk (HR = 1.27, 95% CI: 1.09–1.47, *p* = 0.002), and Model 2 confirmed this finding (HR = 1.31, 95% CI: 1.13–1.52, *p* < 0.001). After full adjustment in Model 3, the connection was reduced and was no longer statistically significant (HR = 1.15, 95% CI: 0.97–1.36, *p* = 0.098). RCS analysis (Figure 2(C)) further suggested a nonlinear relationship between log (CMI) and stroke risk, indicating that the association may not be adequately characterized by a single linear effect estimate. In the DM group, stroke incidence significantly varied among the different CMI quartiles. Compared to Q1, Q4 (HR = 1.49, 95% CI: 1.02–2.31, *p* = 0.019), Q3 (HR = 1.85, 95% CI: 1.21–2.82, *p* = 0.004), and Q2 (HR = 1.61, 95% CI: 1.05–2.48, *p* = 0.031) demonstrated a significantly higher risk in Models 3. Whether continuous variables or quartile categories were used, no notable associations were found between stroke incidence and the Pre‐DM or NGR groups.

**TABLE 3:** Association between CMI and the risk of stroke according to different glucose metabolic statuses.

Characteristic	*N*	Event *N*	Model 1			Model 2			Model 3		
			HR	95% CI	*p* value	HR	95% CI	*p* value	HR	95% CI	*p* value
Pre‐DM											
Log (CMI)	3222	151	1.28	1.02, 1.62	0.034	1.31	1.04, 1.66	0.022	1.06	0.82, 1.36	0.667
Quartile	3222	151									
Q1	1006	36	Reference	Reference		Reference	Reference		Reference	Reference	
Q2	925	47	1.43	0.93, 2.21	0.105	1.48	0.96, 2.29	0.077	1.33	0.86, 2.08	0.200
Q3	799	33	1.16	0.72, 1.86	0.535	1.20	0.74, 1.93	0.458	0.98	0.60, 1.60	0.927
Q4	492	35	2.03	1.27, 3.23	0.003	2.13	1.33, 3.41	0.002	1.43	0.86, 2.38	0.172
DM											
Log (CMI)	3815	246	1.27	1.09, 1.47	0.002	1.31	1.13, 1.52	< 0.001	1.15	0.97, 1.36	0.098
Quartile	3815	246									
Q1	846	34	Reference	Reference		Reference	Reference		Reference	Reference	
Q2	914	57	1.58	1.03, 2.41	0.035	1.65	1.08, 2.53	0.021	1.61	1.05, 2.48	0.031
Q3	968	79	2.07	1.39, 3.10	< 0.001	2.19	1.46, 3.29	< 0.001	1.85	1.21, 2.82	0.004
Q4	1087	76	1.77	1.18, 2.65	0.006	1.92	1.28, 2.90	0.002	1.49	1.02, 2.31	0.019
NGR											
Log (CMI)	943	77	1.20	0.99, 1.47	0.067	1.23	1.00, 1.52	0.055	1.18	0.94, 1.50	0.159
Quartile	943	77									
Q1	143	5	Reference	Reference		Reference	Reference		Reference	Reference	
Q2	156	12	2.21	0.78, 6.28	0.136	1.99	0.70, 5.69	0.199	1.83	0.64, 5.26	0.260
Q3	228	18	2.30	0.85, 6.18	0.100	2.12	0.77, 5.80	0.144	2.02	0.72, 5.65	0.182
Q4	416	42	2.97	1.17, 7.50	0.022	2.84	1.11, 7.30	0.030	2.43	0.90, 6.56	0.079

*Note:* Log (CMI) denotes the log‐transformed CMI and was used in the continuous‐variable analyses; CMI quartiles (Q1–Q4) were defined using the original, untransformed CMI. Model 1: unadjusted; Model 2: adjusted for age, gender, education level, marital status, drinking status, smoking status; Model 3: Model 2 + adjusted for history of hypertension, history of dyslipidemia, history of heart disease, obesity, heart rate, BUN, eGFR.

Abbreviations: BUN, blood urea nitrogen; CI, confidence interval; CMI, cardiometabolic index; DM, diabetes mellitus; eGFR, estimated glomerular filtration rate; HR, hazard ratio; NGR, normal glucose regulation; Pre‐DM, prediabetes mellitus.

To understand how log (CMI) is associated with stroke risk in varying glucose metabolic statuses, multivariable‐adjusted restricted cubic spline analyses were used. In both the Pre‐DM (Figure 2(B)) and NGR (Figure 2(D)) groups, significant overall or nonlinear connections were not detected (Pre‐DM: *p* overall = 0.658, *p* nonlinear = 0.499; NGR: *p* overall = 0.256, *p* nonlinear = 0.322). On the flip side, the DM group revealed a significant nonlinear pattern (Figure 2(C); *p* overall = 0.007, *p* nonlinear = 0.004), with stroke risk climbing at low to moderate log (CMI) levels and flattening out at higher levels. When stratified by glucose metabolism status, the Kaplan–Meier analyses revealed notable differences in cumulative stroke incidence across CMI quartiles for the Pre‐DM, DM, and NGR groups (all log‐rank *p* < 0.0001; Figure 3(B–D)).

### 3.4. Subgroup Analysis

The analysis of CMI as a continuous variable [log (CMI)] showed a significant association with stroke occurrence only among participants free of heart disease (HR = 1.16, 95% CI: 1.02–1.33; *p* = 0.027; Table 4). For additional subgroups classified by sex, age, BMI, and hypertension status, the associations were not statistically significant, and the effect sizes were modest (all *p* ≥ 0.05; Table 4). In the analysis, no statistically significant interactions were identified involving continuous log (CMI) and multiplicative interaction terms (sex: *p*_interaction = 0.287; age: *p*_interaction = 0.130; BMI: *p*_interaction = 0.190; hypertension: *p*_interaction = 0.422; heart disease: *p*_interaction = 0.243; Table 4). When stratified by the BMI, the association between CMI quartiles and stroke risk differed by adiposity status. Participants with a BMI of less than 24 kg/m^2^ showed no statistically significant association across the CMI quartiles (all *p* > 0.05). In contrast, individuals with a BMI of 24 kg/m^2^ or more had a significant association between increased CMI levels and a higher risk of stroke. The risk of stroke was significantly higher for participants in Q4 than in Q1 (adjusted HR = 2.12, 95% CI: 1.13–3.99, *p* = 0.020) (Figure 4). By stratifying based on hypertension status, a significant association was observed between CMI quartiles and stroke risk in individuals with hypertension (Figure 4). Compared with Q1, participants in Q4 had a significantly higher risk of stroke (adjusted HR = 1.61, 95% CI: 1.06–2.46, *p* = 0.026) (Figure 4). In a similar manner, the relationship between CMI and stroke was identified only in participants without heart disease when classified by heart disease status. In this subgroup, participants in Q4 had a significantly higher risk of stroke (adjusted HR = 1.54; 95% CI: 1.09–2.18, *p* = 0.014). Participants with existing heart disease or those without hypertension showed no significant associations (all *p* > 0.05). No significant association was found between the CMI and stroke across sex groups (all *p* > 0.05) (Figure 4). In analyses accounting for age, a significant association was observed among participants aged 60 and above (Q4 vs. Q1: HR = 1.50, 95% CI: 1.01–2.25, *p* = 0.047), but not in those younger than 60 (Figure 4).

**TABLE 4:** Subgroup analysis of the association between log (CMI) and stroke.

Characteristic	*N*	Event *N*	HR	95% CI	*p* value	*p*_interaction_
Gender						0.287
Male	3689	221	1.17	0.99, 1.39	0.073	
Female	4291	474	1.07	0.90, 1.27	0.434	
Age						0.130
< 60 years	4285	474	1.10	0.92, 1.30	0.301	
≥ 60 years	3695	474	1.15	0.97, 1.36	0.100	
BMI						0.190
BMI < 24	4742	233	1.16	0.98, 1.37	0.091	
BMI ≥ 24	3238	241	1.08	0.91, 1.27	0.393	
Hypertension						0.422
Yes	3228	302	1.09	0.94, 1.26	0.278	
No	4752	172	1.18	0.97, 1.45	0.099	
Heart disease						0.243
Yes	932	95	1.03	0.78, 1.35	0.842	
No	7048	379	1.16	1.02, 1.33	0.027	

*Note:* Log (CMI) denotes the log‐transformed CMI and was used in the continuous‐variable analyses; CMI quartiles (Q1–Q4) were defined using the original, untransformed CMI. Model 1: unadjusted; Model 2: adjusted for age, gender, education level, marital status, drinking status, and smoking status; Model 3: Model 2 + adjusted for adjusted for history of hypertension, history of dyslipidemia, history of heart disease, obesity, heart rate, BUN, eGFR, and GSM.

Abbreviations: BUN, blood urea nitrogen; CI, confidence interval; CMI, cardiometabolic index; DM, diabetes mellitus; eGFR, estimated glomerular filtration rate; GMS, glucose metabolic states; HR, hazard ratio; NGR, normal glucose regulation; Pre‐DM, prediabetes mellitus.

**FIGURE 4:**
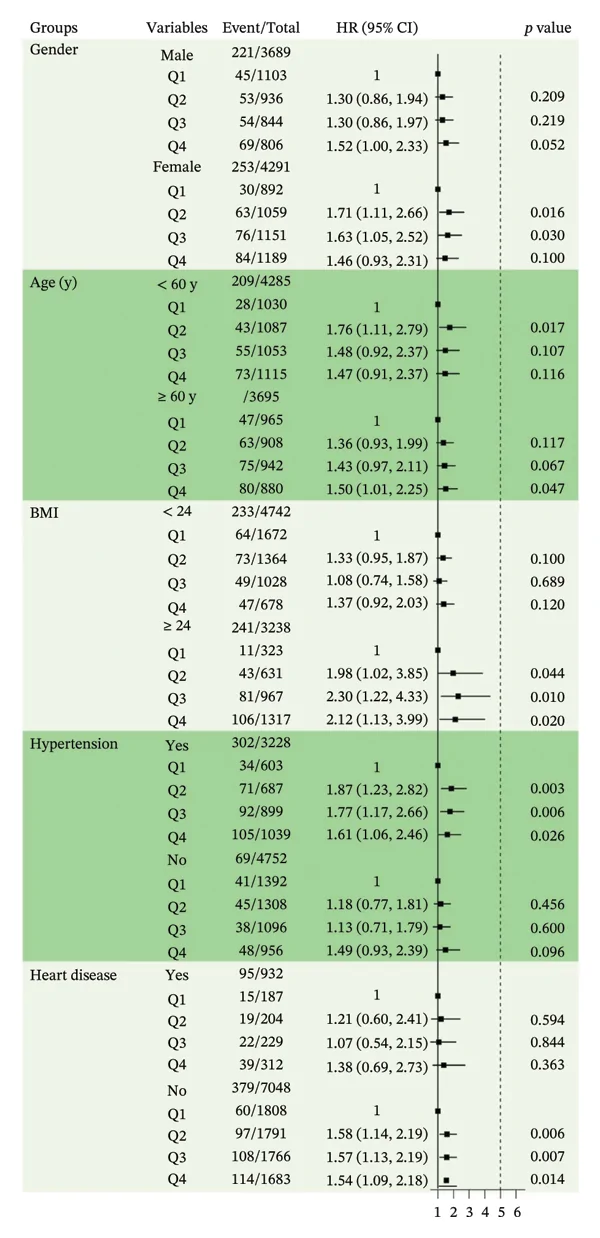
Subgroup analysis of the association between CMI quartiles and stroke risk. *Note:* Log (CMI) denotes the log‐transformed CMI and was used in the continuous‐variable analyses; CMI quartiles (Q1–Q4) were defined using the original, untransformed CMI. Abbreviations: BMI, body mass index; CI, confidence interval; HR, hazard ratio.

### 3.5. Sensitivity Analysis

Multiple sensitivity analyses were performed to evaluate the robustness of the findings. The sensitivity analyses, utilizing Cox regression models, demonstrated a strong positive connection between CMI and stroke risk in the dataset before missing values were addressed through multiple imputation, despite adjustments for potential confounders (Supporting Table 2). In addition, the association between CMI and stroke risk remained statistically significant after adjusting for multiple variables, even after excluding participants who had a stroke in the past 2 years (Supporting Table 3).

### 3.6. Predictive Value of CMI for Stroke Incidence Across Different GMS and Comparison of Its Discriminatory Performance With WHtR and TG/HDL‐C

As depicted in Figure 5, the model’s ability to differentiate varied among the different glucose metabolic categories. The AUC was 0.698 in the Pre‐DM group, 0.675 in the DM group, and 0.693 in the NGR group. Overall, CMI showed modest discriminatory ability for incident stroke across the different glucose metabolic status. In comparing the discriminatory capabilities for incident stroke, ROC curve analysis showed CMI with an AUC of 0.684, slightly exceeding WHtR’s AUC of 0.683 and TG/HDL‐C’s AUC of 0.680 (Figure 6).

**FIGURE 5:**
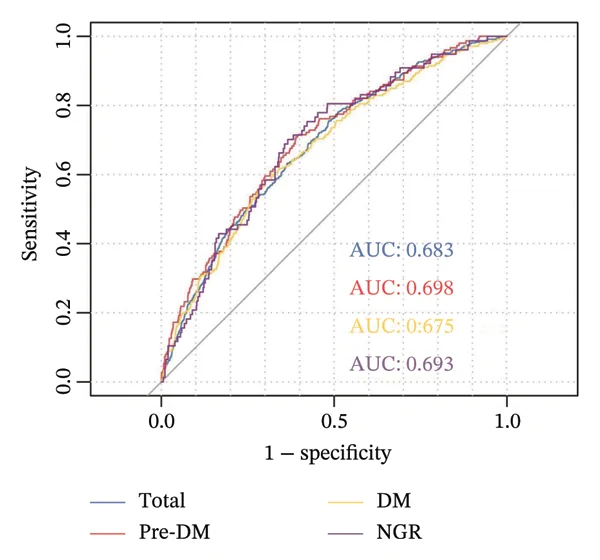
The predictive value of log (CMI) on the risk of stroke under different blood glucose metabolic status. *Note:* Log (CMI) denotes the log‐transformed CMI and was used in the continuous‐variable analyses. Abbreviations: AUC, area under the curve; DM, diabetes mellitus; NGR, normal glucose regulation; Pre‐DM, prediabetes mellitus.

**FIGURE 6:**
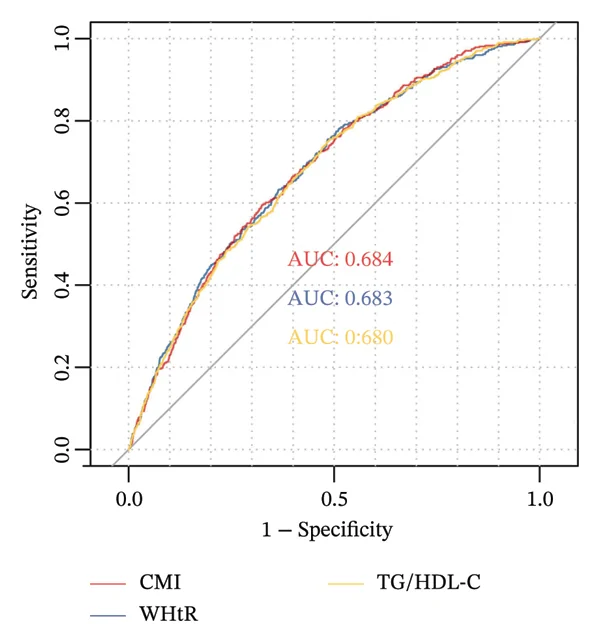
Receiver operating characteristic curves comparing the discriminatory performance of CMI, WHtR, and TG/HDL‐C for incident stroke. Abbreviations: AUC, area under the curve: CMI, cardiometabolic index; ROC, receiver operating characteristic; TG/HDL‐C, triglyceride‐to‐high‐density lipoprotein cholesterol ratio; WHtR, waist‐to‐height ratio.

## 4. Discussion

Data from the CHARLS cohort were used in this longitudinal study to explore the association between the CMI and stroke occurrence and to investigate whether this association varied with glucose metabolism status. Baseline demographic and clinical characteristics showed differences between the CMI quartiles. In the overall cohort, an elevated CMI was connected to a heightened risk of stroke incidents following multivariable adjustments. For participants with DM, increased CMI quartiles were linked to a heightened risk of stroke, unlike in the Pre‐DM or NGR groups where no significant associations were noted. The ROC analysis revealed that CMI’s ability to discriminate incident stroke in those with DM was only modest, with an AUC of 0.675. Although there is a connection between elevated CMI levels and a higher stroke risk in individuals with DM, yet the discrimination was limited. With an overall AUC of 0.684, the CMI was only slightly higher than WHtR (0.683) and TG/HDL‐C (0.680), showing limited extra predictive value beyond its component‐related metabolic indicators. The importance of this association in clinical and preventive contexts requires further confirmation in studies with larger and more ethnically diverse populations.

Obesity, dysglycemia, and dyslipidemia are factors that might affect how the CMI is linked to stroke risk [33]. Various studies have demonstrated a significant correlation between CMI and health issues such as diabetes, hypertension, and dyslipidemia [12, 34, 35]. Furthermore, studies have shown a positive connection between elevated CMI and all‐cause death in older individuals, indicating that CMI could be a significant predictor of unfavorable prognosis in this demographic [36]. Being obese has long been considered a risk factor for experiencing a primary stroke [37–40]. The waist‐to‐height ratio serves as a reliable indicator of obesity, with a focus on central obesity [41, 42]. WtHR has been identified as an important predictor of insulin resistance [43] and can also be used to assess stroke risk [44]. Additionally, a greater weight‐adjusted waist index (WWI) correlates with an increased occurrence of stroke [45]. These results are consistent with our observation that higher CMI levels are related to an increased risk of stroke, but the current observational study cannot determine a causal effect of reducing central obesity or CMI on preventing strokes.

Findings from this study reveal that elevated CMI is an independent risk factor for stroke, in agreement with previous studies that have linked high CMI to stroke risk and prognosis [17]. The original purpose of CMI was to serve as a predictor for diabetes risk. This study is, to the best of our understanding, the first to reveal how the CMI can anticipate stroke risk in individuals with diverse glucose metabolic status. Within the DM group, the connection between CMI and stroke incidents was influenced by the method of CMI modeling. When considering log (CMI) as a continuous variable, the association diminished after full adjustment and failed to reach statistical significance in Model 3 (HR = 1.15, 95% CI: 0.97–1.36; *p* = 0.098). In contrast, dividing the original CMI into quartiles showed that individuals in Q2, Q3, and Q4 had a significantly increased risk of stroke compared to those in Q1. Interestingly, the effect estimates across quartiles did not follow a strictly monotonic pattern, with the highest HR found in Q3 instead of Q4. This pattern hints that the association between the CMI and stroke risk in those with DM might not follow a basic linear dose–response model. This interpretation is further supported by the restricted cubic spline analysis (Figure 2), which demonstrated a significant overall and nonlinear association in the DM group (*p* overall = 0.007; *p* nonlinear = 0.004), with stroke risk increasing at lower‐to‐moderate levels of log (CMI) and tending to plateau at higher levels. The use of a single continuous coefficient for log (CMI) suggests an approximately linear effect over the entire exposure range, possibly weakening associations that differ across various CMI levels. In contrast, quartile‐based analyses relax this linearity assumption and may be more sensitive to threshold‐like or nonlinear patterns. Although limited statistical power cannot be completely excluded as a contributing factor, the significant nonlinear RCS findings suggest that nonlinearity is a more plausible explanation for the discrepancy between the continuous and categorical analyses. Thus, these results should not be interpreted as indicating a simple monotonic dose–response relationship, and further investigations are needed to establish the shape of this association in individuals with DM. However, no meaningful correlation was found between stroke occurrence and either log (CMI) or CMI in the NGR and Pre‐DM groups when considering interquartile groupings. The connection may lie in CMI’s initial purpose as a diabetes risk predictor, suggesting that its predictive capability could be somewhat insufficient for those with NGR and Pre‐DM. Highlighting the need for larger, multicenter studies to further evaluate the association between the CMI and incident stroke across different glucose metabolic status and to validate the robustness and clinical relevance of these findings.

Our observation that the CMI is more closely linked to stroke risk in people with DM might reflect common pathophysiological pathways. To begin with, the CMI covers central obesity and atherogenic dyslipidemia by employing the waist‐to‐height ratio and the TG/HDL‐C ratio, respectively, highlighting two fundamental traits of cardiometabolic dysfunction. Dyslipidemia in obesity is marked by increased TGs, diminished HDL‐C, and small dense LDL particles, all of which promote atherosclerosis [12, 46]. Second, a higher TG/HDL‐C ratio is significantly associated with insulin resistance and impaired endothelial function, both of which lead to vascular damage and the process of thrombogenesis [47]. Third, DM is characterized by chronic hyperglycemia, oxidative stress, and a prothrombotic state with increased platelet reactivity and impaired fibrinolysis, which may amplify the adverse vascular effects associated with an elevated CMI [48, 49]. Fourth, the combined effects of visceral adiposity and inflammation may contribute to atherosclerotic processes and cerebrovascular risk, consistent with recent evidence linking composite indices of visceral adiposity and inflammatory status to stroke [50]. Fifth, the heightened association between the CMI and stroke risk in DM patients might partly result from the interaction between dyslipidemia and the diabetic metabolic context. Insulin resistance and atherogenic dyslipidemia, identified by elevated TG and reduced HDL‐C, are common in DM and may lead to endothelial dysfunction, vascular inflammation, and atherosclerosis. From the perspective of hormones and metabolism, the CMI may signify the convergence of several linked metabolic irregularities. Combining WHtR, a central adiposity marker, with the TG/HDL‐C ratio, CMI serves as an indicator of atherogenic dyslipidemia associated with insulin resistance [12, 47]. Central adiposity, particularly visceral fat accumulation, is closely linked to adipose tissue dysfunction, increased fatty‐acid flux, and inflammatory signaling, all of which may impair insulin action and contribute to disturbances in glucose homeostasis [51]. In contrast, insulin resistance can drive the liver to produce more TGs and change lipoprotein metabolism, leading to the typical rise in TGs and drop in HDL‐C concentrations [47, 52]. These processes, which are interrelated, establish a metabolic basis for the association between CMI and glucose metabolic dysfunction. Supporting this view, recent data from population‐based research have revealed that increased CMI correlates with insulin resistance, prediabetes, and diabetes [14]. Thus, rather than being a direct measure of insulin resistance, the CMI may serve as a practical composite marker that reflects central obesity, atherogenic dyslipidemia, decreased insulin sensitivity, and glucose regulation issues.

The different associations observed across glucose metabolic states may have implications for metabolic risk stratification. According to the study, the connection between the elevated CMI and stroke was clearer in those with DM, but no significant link was found in participants with Pre‐DM or NGR. This pattern suggests that the relevance of CMI to vascular risk may depend on the underlying metabolic milieu. In individuals with established DM, central adiposity, insulin resistance, dyslipidemia, and chronic hyperglycemia frequently coexist, and CMI may therefore identify individuals with a greater clustering of adverse metabolic characteristics. Supporting this view, recent data from two national cohorts revealed that a higher CMI is correlated with a greater incidence of cardiovascular disease among diabetic individuals [18]. In contrast, Pre‐DM represents a heterogeneous intermediate metabolic state, with substantial variation in insulin sensitivity, β‐cell function, adiposity, and the degree of dysglycemia. Importantly, the absence of a significant CMI‐stroke association in our Pre‐DM group should not be interpreted as indicating an absence of vascular risk. An umbrella review of prospective studies demonstrated that prediabetes is associated with increased risks of cardiovascular disease and stroke [53], while a recent meta‐analysis further showed that both prediabetes and insulin resistance are associated with recurrent vascular events after ischemic stroke or transient ischemic attack [54]. The results suggest that metabolic risk could already be present before diabetes is fully developed, although CMI may not capture this risk equally at every stage of glucose dysregulation. Consequently, CMI should perhaps be seen as a complementary marker for metabolic risk, rather than a universal or sole predictor of vascular risk. In studies based on populations, higher CMI has been connected to HOMA‐IR, fasting glucose, HbA1c, prediabetes, and diabetes [14]. By promoting hepatic TG production and causing disturbances in HDL metabolism, insulin resistance may contribute to the high TG and low HDL phenotype detected by the CMI [52]. Once diabetes is established, persistent hyperglycemia, oxidative stress, and inflammatory signaling can aggravate endothelial dysfunction by reducing nitric oxide bioavailability and promoting vascular inflammation and atherosclerotic injury [55, 56]. These abnormalities, together with diabetes‐related conditions that promote atherosclerosis and thrombosis, may enhance the susceptibility to cerebrovascular events [57, 58]. Therefore, the CMI might indicate the merging of metabolic issues related to fat, lipids, and glucose that lead to vascular damage. Nevertheless, these pathways provide biological plausibility rather than evidence of mediation or causality, and mechanistic studies incorporating direct measures of insulin resistance, β‐cell function, and vascular dysfunction are needed to clarify these relationships.

The CMI offers a combined measure of metabolic disorders related to cerebrovascular risk by incorporating central adiposity and atherogenic dyslipidemia. Unlike the BMI, which reflects overall body mass but does not capture fat distribution [59]. CMI incorporates WHtR, a marker of visceral adiposity that has been more strongly associated with cardiometabolic risk than the BMI in several studies [44, 60]. Concurrently, the TG/HDL‐C ratio, which is a component of CMI, represents disturbances in lipid metabolism and insulin resistance, contributing to both atherosclerosis and stroke. Even though indices like TyG and TG/HDL‐C have been tied to stroke risk, they do not directly factor in central fat accumulation [61, 62]. Consequently, the CMI may offer a more unified analysis of metabolic disorders connected to stroke risk.

The findings, derived from a nationally representative group of Chinese adults aged 45 and over, should be cautiously generalized to younger populations and ethnic groups outside of China. Outside of China, the evidence on the CMI related to stroke is limited, with most studies being cross‐sectional in nature. Findings from U.S. cohorts have mainly tied CMI to a wide array of cardiovascular outcomes, including mortality from all causes and cardiovascular issues, as well as existing cardiovascular diseases [63]. Beyond the findings of CHARLS, cross‐sectional studies in rural China also show a positive relationship between CMI and stroke [64]. Together, these results endorse the rationale for stroke‐specific, forward‐looking external validation in younger and ethnically diverse populations, and recalibration if performance shifts in different contexts.

This study’s major strength lies in employing a large, nationally representative longitudinal cohort of middle‐aged and older Chinese adults across various glucose metabolic states. The robustness of the findings was assessed through multiple sensitivity analyses and adjustments for multiple variables. The CMI, which can be calculated from common anthropometric and lipid measurements, could be valuable for evaluating stroke risk in epidemiological research, but its clinical usefulness still needs further confirmation. This research has a number of limitations that should be acknowledged. First, excluding participants who were missing data necessary for CMI calculation or stroke outcome information reduced the analytic sample size and might have introduced selection bias. Although multiple imputation was applied to missing covariate data, we did not impute the primary exposure or outcome because doing so would require additional assumptions and could introduce uncertainty into the estimated exposure‐outcome association. For this reason, the primary exposure and outcome were assessed using a complete‐case approach. The potential systematic differences between excluded and included participants cannot be completely dismissed, which may impact the generalizability of our conclusions. Second, because enrollment required survival until baseline, survivor bias cannot be ruled out, particularly among older adults who may have experienced earlier stroke events and were therefore not represented. Third, the dataset from CHARLS did not include detailed information on the subtypes of strokes. Thus, we were unable to conduct analyses specific to subtypes or determine whether the association between CMI and stroke varied between ischemic and hemorrhagic types. The clinical interpretation of our findings is constrained, and further studies with clinically adjudicated stroke subtypes are needed to clarify whether the observed association is mainly driven by ischemic stroke. Fourth, the determination of stroke outcomes was based on self‐reported physician diagnoses instead of validated clinical events. Even though this approach is broadly used in aging populations and has shown reasonable validity for estimating stroke incidence and exposure–outcome associations, misclassification and recall errors might still occur, especially among older participants. However, because stroke events in CHARLS were not adjudicated against medical records or imaging in our analytic cohort, study‐specific estimates of sensitivity and specificity were unavailable, precluding a reliable quantitative assessment of the magnitude of this bias. Fifth, the assessment of the CMI was conducted only initially and was assumed to be constant over the follow‐up. Relying on a single baseline measurement might have caused exposure misclassification and regression dilution bias, as the CMI and its components may change over time. Time‐varying analyses could not be executed because the repeated measurements required for CMI were not available. Incorporating repeated CMI measurements is recommended for future studies. Sixth, excluding participants, especially those lacking data needed for CMI calculation, might have caused selection bias and decreased the representativeness of the final analysis sample. Although large‐scale longitudinal epidemiological studies often face missing data and participant exclusions, we cannot disregard the possibility that the participants analyzed were systematically different from those excluded. Hence, the applicability of our findings to the larger CHARLS population and other groups should be approached with caution. Finally, even with numerous covariate adjustments, there remains a chance of residual confounding due to factors that are either unmeasured or not precisely captured. When combined, these limitations suggest potential biases in selection (survivor and attrition), information (self‐reported outcomes and misclassification), and confounding (unmeasured variables) and should be taken into account when interpreting our findings. In upcoming research, it is necessary to conduct larger cohort studies with prolonged follow‐up durations to better estimate how CMI quartiles are associated with different stroke subtypes.

## 5. Conclusions

The longitudinal analysis revealed that a higher starting CMI was associated with a greater risk of stroke, with the association being more significant among those with DM. Due to its modest discriminatory performance, CMI is limited as a standalone prediction tool. Additional large‐scale and externally validated studies are essential to elucidate its potential in stroke risk assessment.

## Author Contributions

Meng Li and Yongbo Yu were responsible for the conceptualization and design of the study framework. Data analysis and result interpretation were carried out by Xuan Chen, Yonglai Zhang, and Wei Lu. The draft of the manuscript was written by Yongbo Yu, Yonglai Zhang, and Wei Lu. To boost intellectual rigor and accuracy, Meng Li, Xuan Chen, and Wei Lu provided important revisions.

## Funding

This research was supported by the Science and Technology Planning Project of Taizhou (No. 25YWB103) and the Zhejiang Provincial Administration of Traditional Chinese Medicine Science and Technology Project (No. 2026ZL1018).

## Disclosure

The final manuscript was approved by all authors after a critical review and they collectively accept full responsibility for its integrity and reliability.

## Ethics Statement

The original CHARLS cohort study was approved by the Biomedical Ethics Review Committee of Peking University (IRB00001052‐11015), and written informed consent was obtained from all participants. The present study used publicly available, de‐identified CHARLS data for secondary analysis. Because this analysis involved only anonymized data and did not include additional participant recruitment or interaction, no additional ethical approval or exemption was required.

## Consent

Please see the Ethics Statemnt.

## Conflicts of Interest

The authors declare no conflicts of interest.

## Supporting Information

Additional supporting information can be found online in the Supporting Information section.

## Supporting information


**Supporting Information** Supporting Table 1: Distribution of missing data. Supporting Table 2: The HR (95% CI) of stroke according to CMI in the three models with missing data. Supporting Table 3: The HR (95% CI) of stroke according to the CMI in the three models excluding strokes within 2 years.

## Data Availability

The data that support the findings of this study are available from the corresponding author upon reasonable request.
