# Supplementary material for: Link Between the Cardiometabolic Index and Stroke Risk Across Different Glucose Metabolic Status in Chinese Middle‐Aged and Older Adults: A Longitudinal Cohort Study
Source: Int J Endocrinol. 2026 Oct 13;2026:6169486. doi: 10.1155/ije/6169486 (PMC13660412; doi:10.1155/ije/6169486)
Supplement: Supplementary file 1 [file IJE-2026-6169486-s001.docx]

**Supplementary Table 1** Distribution of missing data

| **Characteristic** | **No. of missing values** | **Percent (%)** |
| --- | --- | --- |
| **Obesity status** | 24 | 0.30 |
| **CMI** | 0 | 0.00 |
| **Age** | 0 | 0.00 |
| **Gender** | 5 | 0.06 |
| **SBP** | 82 | 1.03 |
| **DBP** | 98 | 1.23 |
| **Education** | 2 | 0.03 |
| **Smoking** | 24 | 0.30 |
| **Drinking** | 3 | 0.04 |
| **Heart rate** | 58 | 0.73 |
| **Heart disease** | 24 | 0.30 |
| **Dyslipidemia** | 80 | 1.00 |
| **HDL** | 0 | 0.00 |
| **LDL** | 15 | 0.19 |
| **FPG** | 0 | 0.00 |
| **WC** | 0 | 0.00 |
| **WHR** | 0 | 0.00 |
| **TC** | 0 | 0.00 |
| **TG** | 0 | 0.00 |
| **Serum creatinine** | 5 | 0.06 |
| **BUN** | 2 | 0.03 |
| **eGFR** | 10 | 0.13 |
| **HbA1c** | 0 | 0.00 |
| **GSM** | 0 | 0.00 |

**Abbreviations:**CMI, Cardiometabolic Index; SBP, systolic blood pressure; DBP, diastolic blood pressure; WHtR, Waist to Height Ratio; TC, Total cholesterol; TG, Triglyceride; HDL-C, high-density lipoprotein cholesterol; LDL-C, low-density lipoprotein cholesterol; HbA1c, Hemoglobin A1c; FPG, fasting plasma glucose; eGFR, estimated glomerular filtration rate; GMS, glucose metabolic states; NGR, normal glucose regulation; Pre-DM, prediabetes mellitus; DM, diabetes mellitus

**Supplementary Table 2** The HR (95% CI) of stroke according to CMI in the three models with missing data

| **Characteristic** | **N** | **Event N** | **Model 1** | | | **Model 2** | | | **Model 3** | | |
| --- | --- | --- | --- | --- | --- | --- | --- | --- | --- | --- | --- |
|  |  |  | **HR** | **95% CI** | **P-value** | **HR** | **95% CI** | **P-value** | **HR** | **95% CI** | **P-value** |
| **Log(CMI)** | 7,980 | 474 | 1.32 | 1.19, 1.46 | <0.001 | 1.35 | 1.22, 1.50 | <0.001 | 1.13 | 1.00, 1.28 | 0.044 |
| **Quartile** | 7,980 | 474 |  |  |  |  |  |  |  |  |  |
| Q1 | 2,029 | 77 | Reference | Reference |  | Reference | Reference |  | Reference | Reference |  |
| Q2 | 2,007 | 115 | 1.53 | 1.14, 2.04 | 0.004 | 1.58 | 1.18, 2.12 | 0.002 | 1.50 | 1.11, 2.01 | 0.008 |
| Q3 | 1,977 | 132 | 1.79 | 1.35, 2.37 | <0.001 | 1.88 | 1.42, 2.50 | <0.001 | 1.56 | 1.16, 2.11 | 0.003 |
| Q4 | 1,967 | 150 | 2.05 | 1.56, 2.70 | <0.001 | 2.20 | 1.66, 2.91 | <0.001 | 1.52 | 1.11, 2.08 | 0.008 |
| ***P for trend*** |  |  |  |  | <0.001 |  |  | <0.001 |  |  | <0.001 |

**Model 1:** unadjusted; **Model 2:** adjusted for age, gender, education level, marital status, drinking status, smoking status; **Model 3:** Model 2+adjusted for adjusted for adjusted for history of hypertension, history of dyslipidemia, history of heart disease, obesity, heart rate, BUN, eGFR, GSM.

**Note:** log(CMI) denotes the log-transformed CMI and was used in the continuous-variable analyses; CMI quartiles (Q1-Q4) were defined using the original, untransformed CMI.

**Abbreviation:** CMI, cardiometabolic index; BUN, blood urea nitrogen; eGFR, estimated glomerular filtration rate; GMS, glucose metabolic states; NGR, normal glucose regulation; Pre-DM, prediabetes mellitus; DM, diabetes mellitus; HR: hazard ratio; CI: confidence interval

**Supplementary Table 3** The HR (95% CI) of stroke according to CMI in the three models excluding strokes within 2 years

| **Characteristic** | **N** | **Event N** | **Model 1** | | | **Model 2** | | | **Model 3** | | |
| --- | --- | --- | --- | --- | --- | --- | --- | --- | --- | --- | --- |
|  |  |  | HR**^1^** | 95% CI**^1^** | **P-value** | HR**^1^** | 95% CI**^1^** | **P-value** | HR**^1^** | 95% CI**^1^** | **P-value** |
| **Log(CMI)** | 7,960 | 454 | 1.32 | 1.19, 1.46 | <0.001 | 1.34 | 1.21, 1.49 | <0.001 | 1.14 | 1.01, 1.29 | 0.036 |
| **Quartile** | 7,960 | 454 |  |  |  |  |  |  |  |  |  |
| Q1 | 1,995 | 75 | Reference | Reference |  | Reference | Reference |  | Reference | Reference |  |
| Q2 | 1,988 | 109 | 1.47 | 1.10, 1.98 | 0.010 | 1.51 | 1.12, 2.03 | 0.006 | 1.41 | 1.04, 1.89 | 0.025 |
| Q3 | 1,989 | 124 | 1.68 | 1.26, 2.24 | <0.001 | 1.73 | 1.29, 2.30 | <0.001 | 1.41 | 1.04, 1.91 | 0.025 |
| Q4 | 1,988 | 146 | 1.99 | 1.51, 2.63 | <0.001 | 2.09 | 1.58, 2.77 | <0.001 | 1.45 | 1.07, 1.98 | 0.018 |
| ***P for trend*** |  |  |  |  | <0.001 |  |  | <0.001 |  |  | <0.001 |

**Model 1:** unadjusted; **Model 2:** adjusted for age, gender, education level, marital status, drinking status, smoking status; **Model 3:** Model 2+adjusted for Model 2+adjusted for adjusted for adjusted for history of hypertension, history of dyslipidemia, history of heart disease, obesity, heart rate, BUN, eGFR, GSM.

**Note:** log(CMI) denotes the log-transformed CMI and was used in the continuous-variable analyses; CMI quartiles (Q1-Q4) were defined using the original, untransformed CMI.

**Abbreviation:** CMI, cardiometabolic index; BUN, blood urea nitrogen; eGFR, estimated glomerular filtration rate; GMS, glucose metabolic states; NGR, normal glucose regulation; Pre-DM, prediabetes mellitus; DM, diabetes mellitus; HR: hazard ratio; CI: confidence interval
